# Cari p 1, a Novel Polygalacturonase Allergen From Papaya Acting as Respiratory and Food Sensitizer

**DOI:** 10.3389/fpls.2018.00823

**Published:** 2018-06-18

**Authors:** Moumita B. Sarkar, Gaurab Sircar, Nandini Ghosh, Abhishek K. Das, Kuladip Jana, Angira Dasgupta, Swati G. Bhattacharya

**Affiliations:** ^1^Division of Plant Biology, Bose Institute, Kolkata, India; ^2^Division of Molecular Medicines, Bose Institute, Kolkata, India; ^3^Chest Clinic, Department of Internal Medicine, B. R. Singh Hospital and Centre for Medical Education and Research, Kolkata, India

**Keywords:** papaya, Cari p 1, allergen, pollen fruit cross-sensitization, IgE, polygalacturonase, allergy

## Abstract

Papaya has been reported to elicit IgE-mediated hypersensitivity via pollen inhalation and fruit consumption. Certain papaya sensitive patients with food allergy were found to experience recurrent respiratory distresses even after quitting the consumption of fruits. This observation prompted us to investigate the allergens commonly present in fruits and pollen grains of papaya. A discovery approach consisting of immunoproteomic detection followed by molecular characterization led to the identification of a novel papaya allergen designated as Cari p 1. This allergen was detected as a 56 kDa IgE-reactive protein from pollen as well as fruit proteome through serological analysis. The protein was identified as an endopolygalacturonase by tandem mass spectrometry. Full length Cari p 1 cDNA was isolated from papaya pollen, cloned in expression vector, and purified as recombinant allergen. The recombinant protein was monomeric and displayed pectinolytic activity. Recombinant Cari p 1 reacted with IgE-antibodies of all the papaya sensitized patient sera. In addition to IgE-reactivity, rCari p 1 displayed allergenic activity by stimulating histamine release from IgE-sensitized granulocytes. CD-spectroscopy of rCari p 1 revealed the presence of predominantly β-sheet characters. The melting curve of the allergen showed partial refolding from a fully denatured state indicating the possible presence of conformational IgE-epitopes characteristic of inhalant allergens in addition to the linear IgE-epitopes of food allergens. The expression of this allergen in papaya fruits was detected by immunoblot with anti-Cari p 1 rabbit IgG and reconfirmed by PCR. In an *in vivo* mouse model, rCari p 1 exhibited a comparable level of inflammatory responses in the lung and duodenum tissues explaining the dual role of Cari p 1 allergen in respiratory sensitization via pollen inhalation and sensitization of gut mucosa via fruit consumption. Purified rCari p 1 can be used a marker allergen for component-resolved molecular diagnosis. Further immunological studies on Cari p 1 are warranted to design immunotherapeutic vaccine for the clinical management of papaya allergy.

## Introduction

IgE-antibody mediated type-I hypersensitivity has assumed a pandemic dimension with a prevalence rate of 10–30% of the global population (Pawankar, 2014). A substantial population of the allergy sufferers are sensitized to plant-derived environmental and food allergens. Individuals affected by pollinosis frequently experience ‘pollen-food allergy syndrome,’ popularly known as ‘Oral allergy syndrome’ (OAS). It is a specific type of immediate hypersensitivity caused by cross-reactivity between pollen allergens and homologous plant-derived food proteins (Muluk and Cingi, 2018). This cross-reactivity arises due to the presence of epitopes commonly shared by the tertiary structures of the pollen allergen and the homologous food allergens. In case of OAS, the inflammation is usually restricted to the buccal cavity since the allergens are rapidly degraded by gastric enzymes upon ingestion (Diaz-Perales et al., 1999; Price et al., 2015). About 20–70% individuals with prior sensitization to inhalant pollen grains are reported to suffer from OAS after consuming raw fruits, vegetables, nuts, and certain spices (Flores et al., 2012). On the contrary, the actual food allergens are more resistant to degradation (Besler et al., 2001; Sathe et al., 2005; Moreno, 2007) and are capable of eliciting an IgE-mediated systemic reaction in individuals with food hypersensitivity (Renz et al., 2018).

In all the cases, the efficacy of accurate diagnosis and specific immunotherapy of allergy largely depends on the availability of the molecular and structural details of the allergen molecule. In the past few decades, considerable advancement in recombinant DNA technology (Mothes et al., 2006), bioinformatics (Sircar et al., 2014a,b), and proteomics (Ghosh et al., 2016) has rendered a paradigm shift in the field of molecular allergology. This has enabled the researchers to perform detail characterization of allergens and to synthetically prepare the allergen molecules in large quantity with the desired level of purity. These recombinant allergens are now introduced into component-resolved diagnosis by ImmunoCAP (Schiener et al., 2017) and allergen microarray (Wickman et al., 2017). Furthermore, the hypoallergenic versions of the recombinant allergens have shown promising performance in allergen-specific immunotherapy (SIT) (Valenta et al., 2016). Therefore, the identification, purification, and molecular characterization of allergens are basic criteria for the proper management of allergic disorders.

Papaya (*Carica papaya* L.) of Caricaceae family is globally cultivated for its edible fruits with high nutritious value (Vij and Prashar, 2015). The papaya fruits are consumed either in ripe form simply as fruits or cooked as vegetables at the pre-ripening stage. It is a wind-pollinated plant, and the pollen grains are the primary constituents of ambient bioaerosol (Ghosal et al., 2015). These pollen grains are rich sources of aeroallergens, which can elicit IgE-mediated sensitization of respiratory mucosa leading to symptoms like asthma, allergic rhinitis, and hay fever (Blanco et al., 1998). In addition to pollen, the papaya fruits upon consumption can also lead to atopic sensitization in the gut mucosa with symptoms like oral itching, hives, shortness of breath (Mandal et al., 2009) and even severe gastrointestinal shock (Sharda et al., 2010). Some allergenic components were primarily detected by IgE-serology of the papaya pollen (Chakraborty et al., 2005) and fruit pulp (Iida et al., 2014). In a RAST-based immunochemical investigation, significant cross-reactivity was found between papaya pollen and papaya fruit (Blanco et al., 1998). In the present study, we report an interesting pattern of hypersensitivity in which a polygalcturonase allergen of papaya pollen was found to elicit respiratory symptoms among the papaya food allergic patients even after quitting the consumption of fruits.

## Materials and Methods

### Plant Materials

The dioecious and edible Indian variety of papaya (*Carica papaya* L.), known as ‘Pusa nanha,’ was grown in the experimental garden of Bose Institute, Kolkata, India. The tricolporate pollen grains were isolated (with less than 1% non-pollen contaminants) from the mature inflorescence of the experimental male plants. Fruits were collected from female plants at two different stages of edible form such as pre-ripening (PR) stage (i.e., 30 days post-fertilization; with green peel, white pulp, white seeds, and milky latex) and late ripening (LR) stage (i.e., 75 days post-fertilization; with yellow peel, yellow pulp, black seeds, and watery latex).

### Human Subjects

Residual sera from routine diagnosis of pollen-sensitized patients (*n* = 7) suffering from respiratory allergy (atopic asthma and rhinitis) were collected from the chest clinic of B. R. Singh Hospital and Centre for Medical Education and Research, Kolkata, India. Five of them were reported having mild to moderate allergic reactions to papaya fruits. These patients were diagnosed with an elevated level of specific IgE-antibody against the fruit and pollen extracts of papaya as determined by direct ELISA (Saha et al., 2015). Control sera from patient with either dust mite allergy or mustard allergy and one healthy volunteer were also collected. The clinical and demographic features of the participants are illustrated in **Table 1**. The blood samples were collected with written consents from the subjects. The samples were analyzed in a retrospective and anonymous manner with approval from human ethics committee of the concerned institutes.

**Table 1:** Clinical and demographic features of the human subjects participated in this study.

Sl. No.	Age/Sex	tIgE (IU/ml)	sIgE (PP) (A_405_)	sIgE (PF) (A_405_)	Other sensitizers
P1	44/F	880	1.031	0.820	Ragweed
P2	28/F	1100	0.951	0.943	Mugwort
P3	36/F	843	0.752	0.846	Ragweed
P4	41/M	652	0.621	0.557	Ragweed and Grass
P5	29/F	1200	0.550	0.488	ND
P6	31/F	552	0.542	0.353	ND
P7	51/M	881	0.808	0.780	Ragweed
DM	33/M	1044	0.050	0.013	No
MT	33/M	781	0.113	0.096	No
H	28/M	22	0.032	0.052	No

*Subjects P1–P7 are the patients with allergy to papaya. Patients allergic to dust mite (DM) and mustard (MT) but are not sensitized to papaya (negative controls). Healthy (H) volunteer without elevated level of serum IgE served as negative control. tIgE, total serum IgE; sIgE, specific serum IgE; PP, papaya pollen extract; PF, papaya fruit extract; ND, not determined.*

### Pollen and Fruit Proteome Extraction

About 100 mg of papaya pollen was incubated overnight with 10% TCA in acetone containing 1% DTT. The pellet was washed thrice with chilled acetone containing 1% DTT, and the protein was solubilized in urea buffer (7 M urea, 4% CHAPS, 5 mM EDTA and protease inhibitor cocktail). For papaya fruit proteome, the lyophilized pulp, and peel were homogenized in liquid nitrogen and mixed with extraction buffer (25 mM Tris pH 8.0, 10 mM EDTA, 750 mM sucrose, 25 NP-40, 20 mM KCl and 2% β-ME) for overnight. The supernatant was then mixed with equal volume of tris-phenol (SRL Lab., India) and homogenized on a shaker for 30 min at 4°C. After centrifugation, the upper phenol phase was collected, and the protein was precipitated with 5 volumes of 0.1 M ammonium acetate in methanol. The protein was dissolved in urea buffer. The protein content was estimated by Bradford assay (Biorad), and the quality of extracted protein was checked in 12% SDS-PAGE.

### IgE-Immunoblot

Proteins on PVDF membrane were blocked with 3% BSA and exposed to individual patient sera at 1:10 v/v dilution. The bound IgE-antibodies were detected with monoclonal anti-human IgE secondary antibody with AP conjugate produced in mouse (A3076, Sigma) at 1:1000 v/v and NBT-BCIP (Abcam) substrate (Dey et al., 2016).

### Mass Spectrometry

The 56 kDa band corresponding to the IgE-reactive Cari p 1 band on immunoblot was excised from the SDS-PAGE of pollen protein and was trypsin digested as described in Ghosh et al. (2015). Peptides were processed in ZipTip C18 (Merck Millipore) and analyzed in Autoflex II MALDI-Tof/Tof mass spectrometer (Bruker Daltonics, Germany) under reflector mode equipped with a pulsed nitrogen laser (λ = 337 nm, 50 Hz) at 54% power in the positive ion mode as described earlier in (Sircar et al., 2012). MS/MS was performed with two tryptic peptides having high intensity and suitability (i.e., S/N > 20 and relative isolation) for fragmentation by Laser-Induced Dissociation. The parent ions were selected manually from MS spectra for fragmentation. MS/MS spectra were acquired by laser shots within a range of 4000–8000 using the instrument calibration file. The spectra of parent and fragment ions were analyzed using SNAP algorithm using the FlexAnalysis software (version 3.0, Bruker Daltonics). The processed peaks were transferred through the MS BioTools^TM^ (version 3.4) program as inputs into the MASCOT search engine version 2.4 (Matrix Science, Boston, MA, United States) for protein identification. Spectral baseline subtraction, smoothing, and centroiding were performed. The parameters for MASCOT search include database, NCBInr; taxonomy, Viridiplantae; enzyme, trypsin; universal modification, cysteine carbamidomethylation; variable modification, methionine oxidation; peptide charge state, ‘1+’; missed cleavage, 1; mass tolerance of precursor ions, 100 ppm, and mass tolerance of fragment ions, 0.7 Da. The confidence level of protein identification was based on significant probability score, and minimum 2 unique matched peptides. Protein identification was validated if the peptide ion scores were above the threshold of the significance level of *p* < 0.05, which indicated a 95% confidence level.

### Isolation and Cloning of Cari p 1 cDNA

The total RNA was extracted from freshly harvested papaya pollen following the Trizol method (Invitrogen), and genomic DNA contamination was removed by DNAse-I digestion. The first strand cDNA was synthesized using iScript cDNA synthesis kit (Biorad). The full-length cDNA of Cari p 1 was PCR amplified using gene specific primers (shown in Supplementary Table S1). The cDNA was cloned into pET15b+ (Novogen) vector at NdeI and BamHI sites. The clone was transformed into *Escherichia coli* DH5α strain using LB plates containing 100 μg ml^-1^ ampicillin. Positive transformants containing inserts were confirmed by double digestion. The accuracy of Cari p 1 reading frame was confirmed by Sanger sequencing (Xcelris Genomics, India).

### Purification of Recombinant Cari p 1

The Cari p 1 cDNA clone was transformed into *E. coli* BL21(DE3) (BioBharati LifeScience, India), and the expression of recombinant Cari p 1 (rCari p 1) was induced with 0.5 mM IPTG for 16 h at 20°C. Cells were lysed by sonication in 25 mM Tris-Cl (pH 8.0), 7 M Urea, 300 mM NaCl, 10% glycerol and 10 mM imidazole. The hexahistidine-tagged rCari p 1 was purified by Ni-NTA resin (Qiagen) packed column under the denaturing condition as mentioned in manufacturers protocol. The denatured rCari p 1 was refolded into its soluble form by rapid dilution method (Bhardwaj et al., 2017). Briefly, the Ni-NTA column eluted fraction of purified rCari p 1 was brought to 0.001 mg/ml concentration by rapidly diluting in chilled refolding buffer (50 mM Tris, pH 7.0, 100 mM NaCl, 5% glycerol, 0.5 mM oxidized glutathione, 3 mM reduced glutathione) and the sample was kept on ice for 1 h. The refolded rCari p 1 was then concentrated up to 1.0 mg/ml using Amicon Ultra concentrator device with 10 kDa MWCO (Millipore). The N-terminal hexahistidine tag of rCari p 1 was removed with Thrombin from bovine plasma (Sigma). The purity level of the refolded rCari p 1 was checked by 12% SDS-PAGE (under denaturing and non-denaturing condition). The IgE-reactivity of rCari p 1 was verified by western blot with papaya sensitive patient sera (*n* = 7).

### Endopolygalacturonase Assay

The enzymatic activity of the refolded rCari p 1 was studied by a standard ruthenium red based endopolygalacturonase assay as described by Torres et al. (2011) with slight modification. Briefly, 0.5 μM of rCari p 1 in 80 mM sodium acetate buffer pH 5.5 in a final volume of 100 μl was mixed with increasing amounts of polygalacturonic acid (PGA) in a range of 0 – 0.3% (w/v). The reaction mixtures were incubated at 40°C for 15 min. Following incubation, the volume of the reaction mix was made up to 3 ml with water, and 40 μl of ruthenium red (RR) stock solution (5 mg/ml) was added. The final volume was made up to 6 ml with water, and the absorbance was taken at 535 nm. A calibration curve was prepared with the variable concentration of PGA mixed with RR. Control reactions were prepared without either rCari p 1 or PGA. Enzyme kinetics of rCari p 1 for the hydrolysis of 1 μg of PGA into smaller fragments unable to precipitate with RR per minute was recorded to determine the enzymatic activity.

### Size Exclusion Chromatography

About 1 mg of refolded rCari p 1 was injected into Superdex 200 increase 10/300 GL column connected to AKTA pure FPLC system (GE Lifesciences). The column was calibrated with standard protein mix. The chromatogram was recorded by measuring the A_280_ of the eluted fractions.

### Circular Dichroism Spectroscopy

About 4.8 μM of the refolded rCari p 1 was buffer exchanged with 10 mM NaH_2_PO_4_, pH 7.0. The CD spectra were recorded in a Jasco Corp. J-815 CD spectropolarimeter at 25°C within a wavelength range of 200 – 260 nm. The CD spectra were analyzed using CAPITO server (Wiedemann et al., 2013).

### Degranulation Assay

The allergenic activity of the refolded rCari p 1 was tested by its ability to induce the release of histamine from the IgE-sensitized effector cells following a passive sensitization technique as described in Sircar et al. (2015). Briefly, the granulocytes from a healthy donor were stripped off the bound IgE using 50 mM lactate buffer (pH 3.5). The cells were passively sensitized with either four different patient sera (at 1:10 v/v dilutions) containing high titers of anti-Cari p 1 IgE-antibody or control sera for 120 min at 37°C. The IgE-sensitized cells were then challenged with purified rCari p 1 at a serially increasing concentration ranging from 1.0 to 10000.0 ng/ml. For total release, the cells were lysed with 1% Triton-X. For spontaneous release, cells were not stimulated with rCari p 1. The released histamine in the cell-free supernatant was estimated by EIA Histamine assay kit (Immunotech, Beckman Coulter). The percentage of histamine release was estimated using the following formula:

$$\%\;\text{of}\;\text{release}\;=\;\left(\frac{\text{induced}\;\text{release}\;-\;\text{spontaneous}\;\text{release}}{\text{total}\;\text{release}\;-\;\text{spontaneous}\;\text{release}}\right)\;\times \;100$$

### Detection of Cari p 1 in Papaya Fruit

The refolded rCari p 1 was used to generate polyclonal anti-sera in New Zealand white rabbit following an immunization protocol using Freund’s complete andes) were transferred on PVDF membranes and then exposed to either polyclonal rabbit anti-Cari p 1 antisera or preimmune sera (as a negative control) at 1:100 v/v dilutions. Bound IgG antibodies were detected with anti-Rabbit IgG (whole molecule)–alkaline phosphatase conjugate produced in goat (A3687, Sigma) at 1:30000 v/v dilutions and NBT-BCIP (Abcam) substrate. For reconfirmation at transcript level, the total RNA was extracted from the freshly harvested fruit tissues by SDS-phenol method (Gonzalez-Mendoza et al., 2008) followed by genomic DNA removal and first strand cDNA synthesis. The presence of Cari p 1 transcript was detected by PCR with gene-specific primers.

### Mouse Experiment

The mouse model of papaya allergy was established following an *in vivo* allergen challenge protocol described in Groeme et al. (2016) with certain modifications. Briefly, 6–8 weeks old female BALB/c mice were assorted into four groups (6 mice per group). Two groups of mice were first subcutaneously sensitized with purified rCari p 1 (10 μg antigen/animal) emulsified in Imject Alum Adjuvant (Thermo Fisher Scientific). Following an interval of 7 days after sensitization, one group of mice was challenged with papaya fruit extract via the oral route and the other group was challenged with papaya pollen extract via the intranasal route. The positive and negative control groups were administered with ovalbumin (Sigma) and PBS alone respectively. Finally, the mice were sacrificed after 24 h of the last challenge. The right lung and the gut tissues were sent to a pathological laboratory for histopathological slide preparations following Haematoxylin/Eosin (H/E) staining and Periodic acid Schiff (PAS) staining protocols. The allergen-induced inflammatory changes in the lung and duodenum tissue were recorded under a light microscope and quantified as already described in Sircar et al. (2016).

### Statistical Analyses

Statistical significance (*p <* 0.05) of the inflammatory changes in the histology of Cari p 1 challenged *versus* PBS treated mice was assessed by paired Students’ *t*-test using GraphPad Prism version 6.

## Results

### Allergenic Proteins Commonly Present in Papaya Pollen and Fruit

**FIGURE 1:**
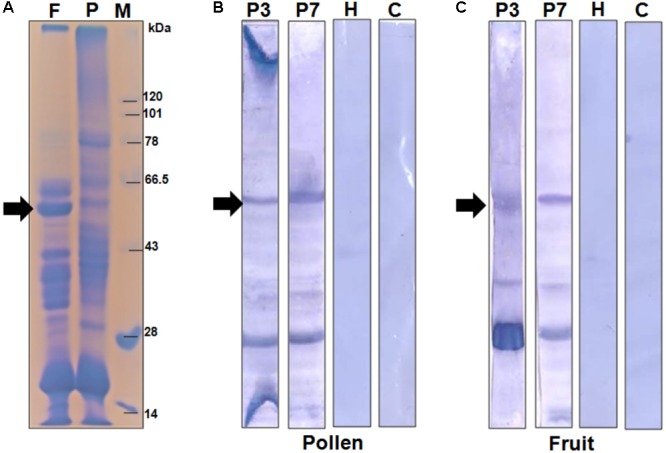
Serological detection of papaya allergens. **(A)** 11% SDS-PAGE of total protein extracted from papaya fruits (lane F), and pollen (lane P). Lane M is protein molecular weight marker. The IgE-immunoblot of pollen protein **(B)** and fruit protein **(C)** with sera from two papaya allergic patients (P3 and P7), one healthy sera (H) and one buffer control i.e., no sera (C). The 56 kDa IgE-reactive is labeled with an arrow.

In the present study, we selected a group of patients who have elevated level of serum IgE-antibody against papaya pollen as well as fruit. These patients reported to experience typical food allergic reactions to papaya fruits. However, the clinical investigations revealed the occurrence of recurrent respiratory symptoms in these patients even after giving up the consumption of papaya fruits. This observation led us to conclude the presence of common allergenic protein(s) in both pollen and fruits of papaya. We, therefore, performed a serological analysis in which the proteomes of papaya pollen and ripe fruit were separately confronted with two of these patient sera to detect the common IgE-reactive component(s). The immunoblot in **Figure 1** displayed two major IgE-reactive bands (mol. wt. ∼56 kDa and ∼30 kDa) commonly present in both pollen and fruit proteome. Hence, these two seroreactive components of papaya pollen and fruits were thought to elicit IgE-mediated hypersensitivity in the respiratory and gut mucosa of these patients respectively. In the present study, we concentrated on the 56 kDa IgE-reactive protein for further identification and characterization at the molecular level.

### A New Endopolygalacturonase Allergen From Papaya

**FIGURE 2:**
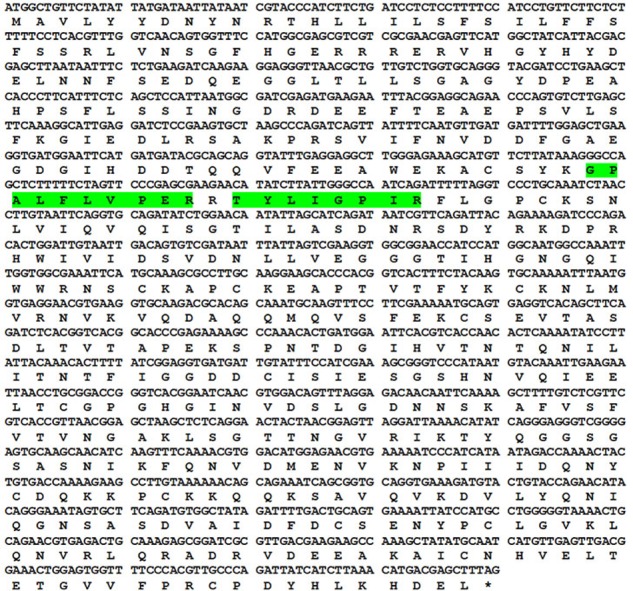
Sequence information of Cari p 1. Codon wise alignment of full length cDNA with corresponding amino acids of the Cari p 1 protein. Two peptides identified by tandem mass spectrometry are highlighted in green.

Using proteomic tools, the 56 kDa protein was identified as an endopolygalacturonase. Searching the MALDI-TOF/TOF spectra against the Viridiplantae NCBI database resulted in the identification of two different peptides of ripening-induced polygalacturonase-2 from *Carica papaya* (**Table 2** and Supplementary Figure S1). These two peptides with m/z 932.54 (TYLIGPIR) and m/z 1098.62 (GPALFLVPER) displayed the significant ion scores of 54 and 44 respectively. The concordance between the theoretical and observed molecular weight further validated the identification process. Both the peptides account for 3.6% of the full length protein. The identified papaya allergen was given the official name Cari p 1 by IUIS. The respective position of the identified peptides in Cari p 1 sequence is shown in **Figure 2**.

**Table 2:** Identification of the 56 kDa papaya allergen by MALDI-Tof/Tof.

Unique peptides	m/z	Protein name	Organism	NCBI Acc.	Ion score	*E* value
RTYLIGPIRF	932.5420	Ripening induced poly-galacturonase 2	*Carica papaya*	gi| 258640138	54	0.0089
KGPALFLVPERR	1098.6080				44	0.082

### Characterization of Recombinant Cari p 1

**FIGURE 3:**
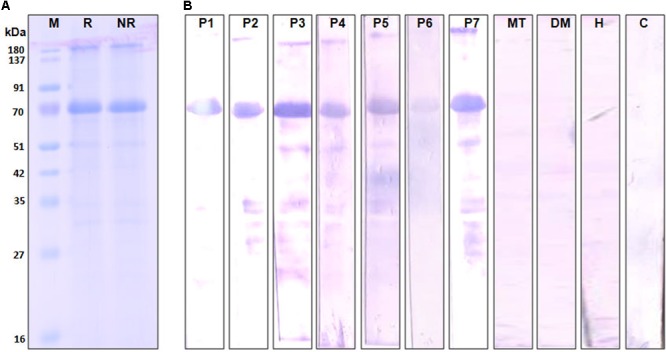
Immunoreactivity of recombinant Cari p 1. **(A)** Purified rCari p 1 in 12.5% SDS-PAGE under reducing (R) and non-reducing (NR) conditions. **(B)** IgE-immunoblot of purified rCari p 1 with sera from seven papaya allergic patients (P1–P7). Negative controls are patient serum with food allergy to mustard (MT), patient serum with respiratory allergy to dust mite (DM), healthy sera (H) and buffer control (C).

The complete amino acid sequence of Cari p 1 allergen was searched in NCBI database using the tBLASTn program, and the full-length cDNA of Cari p 1 (NCBI Acc. No. GQ479794) was retrieved as shown in **Figure 3**. Using gene specific primers, the 1.5 Kb long transcript of Cari p 1 was PCR-amplified and cloned into a suitable expression vector. The rCari p 1 was expressed in inclusion bodies of *E*. *coli* and therefore purified under denaturing condition (**Figure 3A**). The yield of rCari p 1 was about 4 mg L^-1^ of culture. The denatured rCari p 1 was refolded into a soluble form followed by hexahistidine tag removal for downstream immunobiochemical studies. The immunoreactivity of rCari p 1 was checked by western blot with seven individual sera from papaya allergic subjects as shown in **Figure 3B**. The rCari p 1 displayed strong binding with serum IgE-antibodies of all the patients suggesting this protein as an important allergen of papaya. No reactive bands were observed in either control serum or buffer control blot.

### rCari p 1 Is a Monomeric Endopolygalacturonase

**FIGURE 4:**
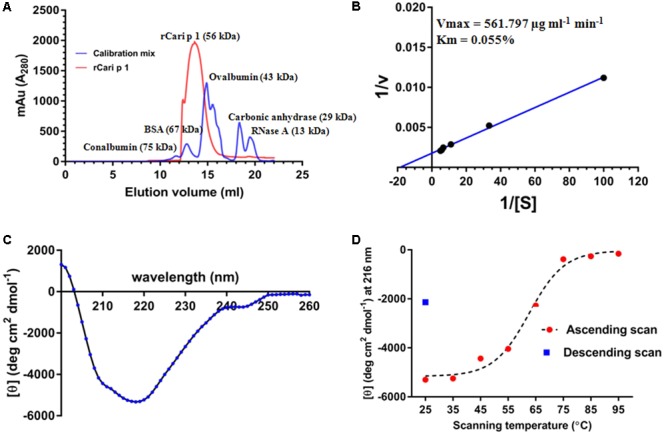
Characterization of rCari p 1: **(A)** Gel filtration chromatogram (red) showing the elution profile of purified ∼56 kDa rCari p 1 as monomer. A mix of five commercially available standard proteins was used for calibration (blue). The *y*-axis denotes the A_280_ of the eluted fractions (mili absorption unit; mAu) and the elution volumes (ml) of the proteins from the size exclusion column are in x-axis. **(B)** Lineweaver–Burk double reciprocal plot showing enzyme kinetics of polygalacturonase (PG) activity of rCari p 1 for polygalacturonic acid (PGA). Purified rCari p 1 was incubated with increasing concentrations (i.e., [S] in %) of PGA (plotted as 1/[S] in *x*-axis) at 40°C for 15 min. PG activity (i.e., v in μg ml^-1^ min^-1^) is calculated as amount of PGA degraded by rCari p 1 allergen per ml of the reaction volume per min (plotted as 1/v in *y*-axis). Activity values are presented as the means of triplicate assay. Displayed are the kinetic parameters (maximum velocity or V_max_ and Michaelis constant or Km) calculated from axis intercepts. **(C)** The far-UV CD spectra of purified rCari p 1, expressed as mean residue molar ellipticity [𝜃] in *y*-axis, recorded in a wavelength range (*x*-axis) at 25°C. **(D)** Melting curve showing gradual changes in ellipticity signal at 215 nm ([𝜃] at λ_max_; *y*-axis) due to rCari p 1 unfolding with increasing temperatures (ascending scan) from 25 to 95°C (*x*-axis). After thermal denaturation, rCari p 1 partially refolded (certain increase in ellipticity signal) upon cooling down (descending scan) from 95 to 25°C.

The aggregation status of rCari p 1 was checked by applying gel filtration chromatography as shown in **Figure 4A**. Under physiological condition (pH 7.2), rCari p 1 eluted from the column as a single fraction with an apparent molecular weight of 56 kDa as determined from the calibration curve of standard mix shown in Supplementary Figure S2. This was reconfirmed in non-reducing SDS-PAGE, where rCari p 1 appeared as a single band and any band corresponding to di/trimeric aggregation was not observed (see **Figure 3A**). The ruthenium red based enzyme assay was performed with 0.16 μM of rCari p 1 to confirm whether the putative allergen retained the functional activity. Purified rCari p 1 showed substrate specificity toward PGA. Typical enzyme kinetics for increased concentration of PGA was observed and plotted according to Lineweaver–Burk shown in **Figure 4B**. The V_max_ and K_m_ of the substrate-saturated enzyme reaction were determined to be 561.7 μg ml^-1^ min^-1^ and 0.055% respectively.

### Folding Pattern and Refolding Behavior of rCari p 1

The CD spectra of rCari p 1 (**Figure 4C**) showed a properly folded protein with predominantly β-sheet characters as the minima were obtained at 215 nm. The denaturation pattern and refolding behavior of rCari p 1 were studied by generating a melting curve (**Figure 4D**) of the protein with increasing temperature. It was observed that the denaturation took place at ∼62°C and the protein became unfolded entirely at 75°C. After cooling back the system to 25°C, a certain increase in the CD signal at λ_215_ was observed indicating a partial refolding of rCari p 1 from a fully denatured state (i.e., partially reversible denaturation).

### rCari p 1 Displayed Allergenic Activity

**FIGURE 5:**
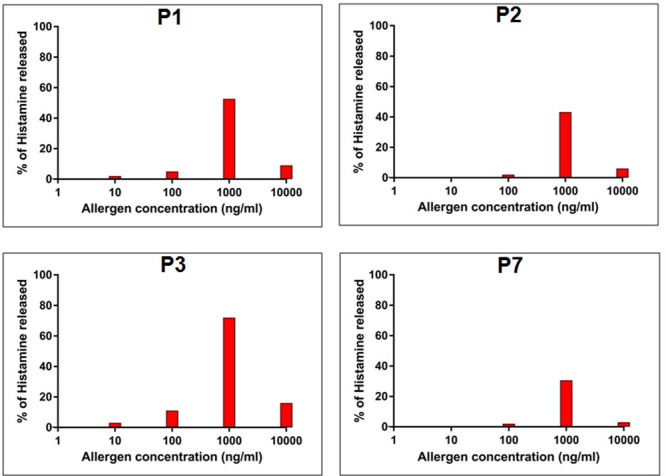
Allergenic activity of rCari p 1. Percentage of histamine release (*y*-axis) from granulocytes passively sensitized with four papaya allergic sera (P1-P3, P7) having high serum IgE-titer against rCari p 1. The cells were stimulated with increasing concentrations (ng/ml) of purified rCari p 1 (*x*-axis).

In addition to IgE-reactivity, the biological activity of rCari p 1, in terms of its allergenicity, was tested by degranulation assay. The rCari p 1 displayed an efficient basophil activation property leading to histamine release by degranulation. Due to unavailability of fresh uncoagulated blood, a passive sensitization protocol was followed. Firstly, the prebound IgE-antibodies were stripped off the FC𝜀R receptors on basophils of a healthy volunteer, and this was followed by addition of IgE-antibody present in the papaya sensitive patient sera. These IgE-sensitized effector cells upon stimulation with rCari p 1 resulted in a dose-dependent release of histamine (**Figure 5**). The highest percentage of degranulation was observed at a concentration of 1000 ng/ml in which the histamine release took place within a range from 30 to 72% (i.e., 50 ± 9.2%) among the four patients tested. Further increasing the allergen concentration (i.e., 10,000 ng/ml) led to a sharp decrease in histamine release. No release was observed with control sera as shown in Supplementary Figure S3.

### Presence of Cari p 1 Was Detected in Papaya Fruits

**FIGURE 6:**
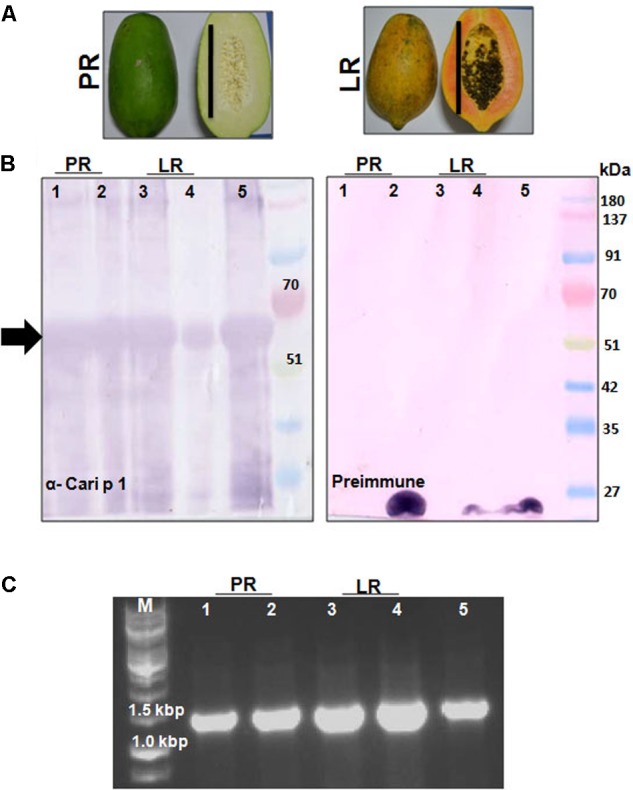
Detection of Cari p 1 allergen in papaya fruits: **(A)** Papaya fruits harvested in two edible stages of fruit maturation such as pre-ripening stage (PR) and late ripening stage (LR). Each bar represents 15 cm length. **(B)** Immunodetection of Cari p 1 allergen in the proteome extracted from the pulp (lane 1 and 3), peel (lane 2 and 4) and pollen grains (lane 5 as positive control) by IgG-western blot with either rCari p 1 specific rabbit antisera (α-Cari p 1) or preimmune sera as negative control. **(C)** 1.2% agarose gel showing PCR based detection of Cari p 1 transcripts in the pulp (lane 1 and 3), peel (lane 2 and 4) and pollen grains (lane 5 as positive control) using gene specific primers.

The polyclonal rCari p 1-specific rabbit antisera was used to screen for the presence of this allergen in fruit tissues at two different maturation stages (**Figure 6A**) by immunoblot. As shown in **Figure 6B**, Cari p 1 was detected in the peel and pulp proteins of papaya fruits at two different harvesting stages, i.e., PR and LR stage. No reactive bands were detected in immunoblot with corresponding preimmune sera. In addition to protein level, the presence of Cari p 1 transcripts were also confirmed in the papaya fruits by gene-specific PCR as shown in **Figure 6C**.

### rCari p 1 Exhibited Respiratory, and Food Allergic Reactions in Mouse Model

**FIGURE 7:**
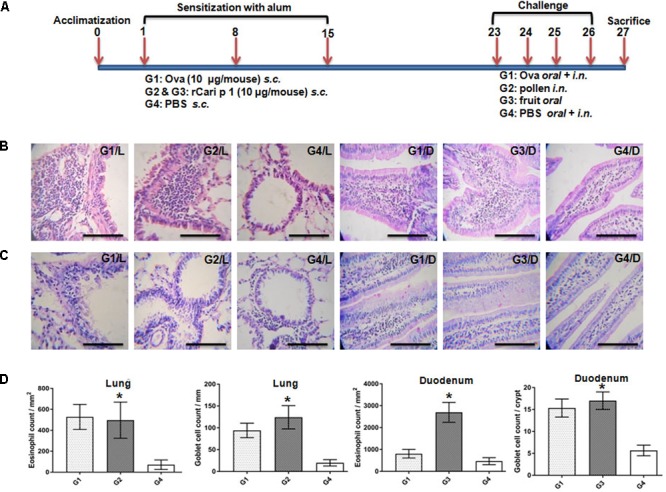
Role of rCari p 1 in mouse model of papaya induced respiratory and food allergy: **(A)** Protocol for mouse model of allergen challenge. Representative histology of mouse lung; L and duodenum; D showing inflammatory changes by H/E staining **(B)** and mucus secretion by PAS staining **(C)**. The bars represent 100 micron length. **(D)** Quantification of allergen-challenge associated inflammatory changes in terms of eosinophil infiltration (mean of eosinophil count ± SD as error bar; *y*-axis) in peribronchial spaces (mm^2^) of lung and lamina propria (mm^2^) of duodenum. Quantification of allergen-challenge associated mucus production in terms of goblet cell hyperplasia (mean of mucus secreting goblet cell count ± SD as error bar; *y*-axis) in bronchial basement membrane (mm) of lung and each crypt of duodenum. Asterisks represent *p* < 0.05 in allergen challenged *versus* PBS treated mice. Six BALB/c mice were taken for each of the four challenge groups (G1 to G4).

The IgE-mediated sensitization potential of rCari p 1 was studied *in vivo* in a mouse model of papaya allergy. The allergen challenge protocol for mice is shown in **Figure 7A**. The rCari p 1 sensitized mice were separately challenged with papaya pollen and papaya fruits via inhalation and ingestion respectively. Allergen-induced eosinophilic inflammations (**Figure 7B**) and mucus secretions (**Figure 7C**) were observed in the lung and duodenum tissues of the mice after nasal and oral challenge respectively. The degree of lung inflammation was quantified in terms of eosinophil infiltration in the peribronchial spaces and mucus-secreting goblet cell proliferation inside the bronchial basement membrane. The degree of gut inflammation was quantified in terms of eosinophil infiltration in the lamina propria and mucus-secreting goblet cell proliferation inside the epithelial tissue of duodenum (**Figure 7D**). The histopathological evidences revealed that rCari p 1 induced inflammatory changes in respiratory and gut mucosa were almost comparable (i.e., no significant differences in cell count) to the corresponding tissue sections of ovalbumin challenged mice. The results clearly demonstrate the dual role of Cari p 1 as a potent respiratory as well as food allergen.

## Discussion

The role of papaya plant in causing IgE-mediated hypersensitivity was studied by several workers from different parts of the world as described elsewhere (Flindt, 1978; Baur et al., 1982; Bernstein et al., 1984; Mansfield et al., 1985; Dando et al., 1995; Soto-Mera et al., 2000). However, till now, no substantial evidence exists to infer the molecular nature of the allergy eliciting components of papaya. The present study, to the best of our knowledge, is the first comprehensive report in which we have cloned the full length cDNA of an important papaya allergen designated as Cari p 1.0101 and performed a detailed immunobiochemical characterization of the recombinant allergen. Our study is a discovery approach in which the clinical manifestations of the hypersensitive reactions in certain papaya allergic patients prompted us to investigate a striking pattern of allergen sensitization. These fruit allergic patients were reported to have papaya plantations in the surroundings of their residences. These patients also informed having significant improvement in food allergic symptoms after quitting the consumption of papaya fruits. However, the occurrences of episodic respiratory allergy in these patients led us to hypothesize the presence of a common elicitor allergen (either identical or structurally similar) in the papaya pollen and fruits. IgE-serology of the pollen and fruit proteome revealed the presence of two major IgE-reactive proteins of different molecular weights. For the present study, we selected the 56 kDa allergen for downstream characterizations. The allergen was identified as an endopolygalacturonase. Plant endopolygalacturonases are pectin degrading enzymes involved in fruit ripening through cell wall maceration (do Prado et al., 2016) and in pollen tube germination (Carvajal et al., 2014). These biological activities clearly substantiate the relevance of Cari p 1 expression in papaya pollen and fruits. The pollen-food cross-reactivity is a unique phenomenon in which IgE-sensitization via a pollen allergen can lead to inflammation of gut mucosa via ingestion of a structurally similar food allergen (often designated as group II food allergen) (Valenta et al., 2015). Such cross IgE-reaction has been well exemplified in the birch pollen allergen Bet v 1 with its structural homolog Mal d 1 from apple and Api g 1 from celery (Haka et al., 2015). Interestingly, in case of papaya, a similar situation was found to arise due to the simultaneous presence of the Cari p 1 allergen in pollen and fruit tissue.

Till now, the worldwide diagnosis of papaya allergy still relies on the use of outdated, less-characterized, and less-standardized crude extracts. In our study, rCari p 1 was found to retain its immunoreactivity as well as allergenic activity. Therefore, the recombinantly purified functional allergen can be considered as a suitable candidate for component-resolved diagnosis. The presence of conserved endopolygalacturonase like catalytic domain in Cari p 1 sequence was validated by enzyme assay for pectinolytic activity similar to an earlier reported plane tree allergen Pla a 2 (Ibarrola et al., 2004). The biologically active rCari p 1 allergen remained as monomer like previously reported polygalacturonase allergen Phl p 13 from grass pollen (Swoboda et al., 2004). The melting curve of rCari p 1 exhibited an interesting pattern of refolding behavior similar to that of the birch pollen allergen Bet v 1 (Laffer et al., 2003). The partial acquisition of native conformation of the heat-denatured rCari p 1 indicated the possibility of conformational IgE-epitope, which can be attributed to the respiratory and oral allergy symptoms reported from the papaya sensitized patients (Pomés, 2010). Since the recombinant Cari p 1 was prepared from pollen grains, so we searched for its presence in the fruit tissues by immunoscreening with rabbit antisera. As expected, the expression of Cari p 1 was detected in the edible pulp and the peel tissues in the advanced stage of fruit maturation. The expression of Cari p 1 was also detected in the fruit at the pre-ripening stage, which is usually consumed as a vegetable. In order to rule out the possibility of non-specific binding due to the polyclonal nature of the anti-Cari p1 rabbit sera, the expression of this allergen in papaya fruits was reconfirmed at the transcript level. The results suggested a wide range of tissue distribution of Cari p 1 in papaya similar to other plant polygalacturonases (Xiao et al., 2017). An almost similar case was observed in an olive pollinosis patient experiencing oral allergic symptoms to olive fruits (Racil et al., 2015). Such a condition can be correlated with the simultaneous presence of Ole e 13 allergen (a thaumatin like protein) in the olive pollen as well as in fruit (Torres et al., 2014). The biological potential of rCari p 1 as a food and respiratory allergen was further reinforced in a murine model. In the *in vivo* model, rCari p 1 displayed its effective role by eliciting inflammatory changes in respiratory as well as in gastrointestinal mucosa.

Taken together, our present study reports Cari p 1 as the first major allergen of papaya. The dual role of Cari p 1 as ingestant and inhalant allergen makes it an ideal candidate for molecular diagnosis of papaya mediated allergic disorders. Further antigenic analyses of Cari p 1 for mapping its immunodominant epitopes may lead to the design of immunotherapeutic vaccines for effective disease management.

## Ethics Statement

This study was carried out in accordance with the recommendations of ‘ICMR guidelines for research involving Human Participants, Bose Institute Human Ethics Committee’ with written informed consent from all the subjects. All subjects gave written informed consent in accordance with the Declaration of Helsinki. The protocol was approved by the ‘Bose Institute Human Ethics Committee.’ This study was carried out in accordance with the recommendations of ‘The Committee for the Purpose of Control and Supervision of Experiments on Animals (CPCSEA) guidelines, Bose Institute Animal Ethics Committee’. The protocol was approved by the ‘Bose Institute Animal Ethics Committee’.

## Author Contributions

GS and MS conceived the study and designed the experiments under the supervision of SB. MS, GS, NG, AD, KJ, and AD performed the experiments. MS and GS analyzed the data. GS, MS, and SB wrote the paper. All the authors have seen and approved the final version of the manuscript.

## Conflict of Interest Statement

The authors declare that the research was conducted in the absence of any commercial or financial relationships that could be construed as a potential conflict of interest.
