# Supplementary material for: Cari p 1, a Novel Polygalacturonase Allergen From Papaya Acting as Respiratory and Food Sensitizer
Source: Front Plant Sci. 2018 Jun 18;9:823. doi: 10.3389/fpls.2018.00823 (PMC6016011; doi:10.3389/fpls.2018.00823)
Supplement: Supplementary file 1 [file Data_Sheet_1.DOCX]

**Cari p 1, a Novel Polygalacturonase Allergen from Papaya Acting as Respiratory and Food Sensitizer**

Moumita Biswas Sarkar^1¥^, Gaurab Sircar^1¥*^, Nandini Ghosh^1^, Abhishek Kumar Das^1^, Kuladip Jana^2^, Angira Dasgupta^3^, Swati Gupta Bhattacharya^1*^

***^1^****Division of Plant Biology, Bose Institute, Kolkata, Pin 700009, India*

***^2^****Division of Molecular Medicines, Bose Institute, Kolkata, Pin 700052, India*

***^3^****Chest Clinic, Department of Internal Medicine, B. R. Singh Hospital and Centre for Medical Education and Research, Kolkata, Pin 700014, India*

¥ Equal Contributions

*Correspondence: Gaurab Sircar, [sircar.gaurab129@gmail.com](mailto:sircar.gaurab129@gmail.com); Swati Gupta Bhattacharya, [swati@jcbose.ac.in](mailto:swati@jcbose.ac.in)

**Supplementary Table**

Table S1: Primers used for amplification of Cari p 1 cDNA

| **Primers** | **Sequences** | **RE sites**  **(underlined)** | **Annealing Temp.** |
| --- | --- | --- | --- |
| Forward | 5’ATGTCATATGATGGCTGTTCTATATTATGA3’ | NdeI | 58ºC |
| Reverse | 5’CGTTGGATCCCTAAAGCTCGTCATGTTTAA3’ | BamHI |  |

**Supplementary Figures**

Figure legends:

Fig. S1: MS/MS spectra with the sequence tag labelled (upper panel) and corresponding screenshots of the MS/MS ion search results in BioTools^TM^ Version 3.4 (lower panel) of two tryptic peptides of Cari p 1.

Fig. S2: Determination of Cari p 1 molecular weight by Gel filtration chromatography using a calibration curve of five standard proteins of known molecular weights. The curve was prepared with K_av_ (y-axis) vs. Log(*MW*) for each protein.

Fig. S3: Histamine release from granulocytes sensitized with control sera (H; healthy serum and DM; dust mite sensitive serum).

**Figure S1**

**
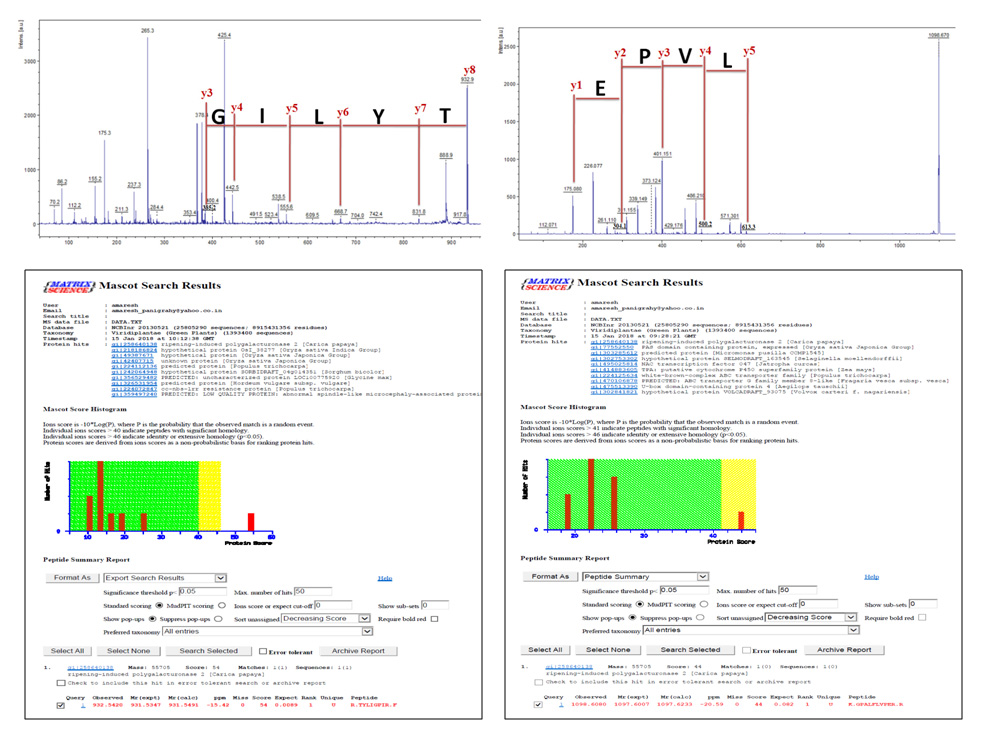
**

**Figure S2**

**
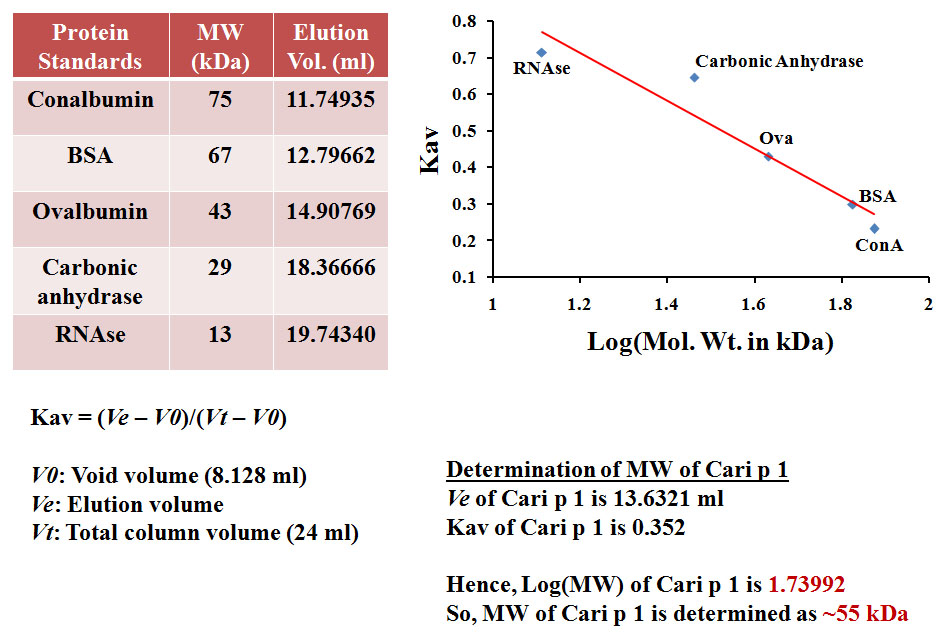
**

**Figure S3**

**
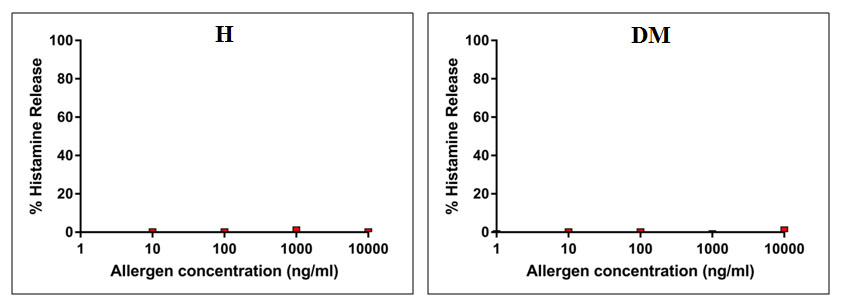
**
